# The Metabolic Stability of the Nicotinic Acetylcholine Receptor at the Neuromuscular Junction

**DOI:** 10.3390/cells10020358

**Published:** 2021-02-09

**Authors:** Isabel Martinez-Pena y Valenzuela, Mohammed Akaaboune

**Affiliations:** 1 Department of Molecular, Cellular and Developmental Biology, University of Michigan, Ann Arbor, MI 48109, USA; isabelm@umich.edu; 2 Program in Neuroscience, University of Michigan, Ann Arbor, MI 48109, USA

**Keywords:** AChR, metabolic stability, synaptic activity, denervation, endocytosis, recycling, degradation

## Abstract

The clustering and maintenance of nicotinic acetylcholine receptors (AChRs) at high density in the postsynaptic membrane is a hallmark of the mammalian neuromuscular junction (NMJ). The regulation of receptor density/turnover rate at synapses is one of the main thrusts of neurobiology because it plays an important role in synaptic development and synaptic plasticity. The state-of-the-art imaging revealed that AChRs are highly dynamic despite the overall structural stability of the NMJ over the lifetime of the animal. This review highlights the work on the metabolic stability of AChRs at developing and mature NMJs and discusses the role of synaptic activity and the regulatory signaling pathways involved in the dynamics of AChRs.

## 1. Introduction

The clustering and maintenance of nicotinic acetylcholine receptors (AChRs) at high density in the postsynaptic membrane is a hallmark of the mammalian neuromuscular junction (NMJ) [1,2,3]. AChRs are transmembrane ligand-gated ion channels, composed of five protein subunits [4]. In the unborn, the composition of AChR consists of α_2_βγδ (immature form) and within a very short time after birth, γ-subunits are replaced by the adult specific ε-subunits to yield the mature AChRs α_2_βεδ. TheS switch in receptor subunits is accompanied by changes in electrophysiological properties of the receptor channel (changes in conductances and gating properties) [5,6,7,8,9,10]. Interestingly, the conversion of receptor channels appears to be tightly regulated by a muscle-specific differentiation program [11]. It should be noted that during the first stages of muscle development, although some receptors form spontaneous clusters, the majority of AChRs are distributed uniformly throughout the membrane of the muscle cells [1,12] and as the development proceeds, AChRs become highly concentrated at the synaptic sites [1,2]. The binding of the neurotransmitter acetylcholine to receptors results in the depolarization of the postsynaptic membrane, leading to muscle contractions [3].

The underlying mechanisms involved in the initial events of AChRs clustering, synapse formation, maturation, and stability have been extensively studied (see reviews [1,2,3]). Briefly, two major groups of proteins have been identified: proteins required for the initial AChRs clustering and synapse formation (the signaling pathway involving agrin, low-density lipoprotein receptor-related protein (LRP4), muscle-specific tyrosine kinase (MuSK), dedicator of cytokinesis family member (Dok7) and rapsyn) and the auxiliary proteins involved in the maturation and stability of NMJs (dystrophin-glycoprotein complex (DGC), neuregulin signaling molecules, Wnt proteins, nonkinase muscle-specific protein) [1,2,3,13,14]. In mice deficient in any of the core proteins AChR clusters simply fail to form, while in mice deficient in auxiliary proteins such as α-dystrobrevin or α-syntrophin, NMJs are formed normally but mature abnormally [15,16,17,18,19].

The nicotinic AChR (a critical downstream signaling transducer in muscle cells) is also required for the clustering and accumulation of the core and auxiliary proteins at synaptic clusters. For instance, targeted elimination of receptor clusters on cultured myotubes with an argon laser not only induced the removal of receptor-associated proteins but also prevented the insertion and accumulation of newly synthesized proteins at the illuminated spots [20]. Additional in vivo studies have shown that AChRs are critical for the targeting of the associated scaffold protein to the synaptic membrane. For example, when the rapsyn coiled-coil (CC) domain, a required domain for the interaction between AChRs and rapsyn was deleted, the targeting of rapsyn to the postsynaptic membrane of NMJs was prevented [21]. Similarly, in zebrafish mutants lacking the expression of AChRs, rapsyn failed to localize at synapses [22,23]. In mice deficient in either fetal or epsilon AChR subunit, the rapsyn expression at the synapse was significantly reduced [24,25] as it was reduced in mice deficient in α-syntrophin and α dystrobrevin, which exhibit a dramatic reduction in total AChR levels compared with wild-type [15,16]. These observations suggest that there is a reciprocal relationship between AChRs and their associated proteins. 

## 2. Turnover Rates of Achrs at Aneural, Developing and Mature NMJS

The use of α-bungarotoxin (BTX) and its derivatives have been instrumental in studying the metabolic stability of AChRs [26,27,28,29,30,31]. The consensus is that AChRs are highly stable in the postsynaptic membrane of a mature NMJ until they are removed and targeted for degradation. When fluorescent or radiolabeled BTX was used as a ligand to monitor the turnover rate of receptors, the half-life was quite long (t _½_ ≈ 9–14 days) at fully functioning synapses, and that this half-life was significantly reduced at surgically denervated synapses (t_1/2_ ≈ 1–3 days) or when synaptic activity was compromised by diseases [32,33,34,35,36]. These estimates were based on the assumption that once AChRs are internalized, they are targeted for degradation (presumably in lysosomes) and did not consider the possibility that those receptors can continuously recycle back to the postsynaptic membrane with their BTX tag.

In 2005, with the development of the sequential method of labeling of AChRs with biotin-bungarotoxin and streptavidin-fluorophore conjugates, it was demonstrated that a significant number of internalized AChRs were able to recycle back to synaptic sites [35,37,38]. This method allows us to distinctly visualize three distinct receptor pools at the NMJ: recycled, pre-existing, and newly synthesized receptors [37]. Of note, it is well established that AChR pools have the same binding affinities for biotin-bungarotoxin/streptavidin. At functional NMJs, changes in fluorescence intensities of recycled and pre-existing AChRs at the same synapses over time showed that the lifetime of recycled receptors is far shorter (t_1/2_ ≈ 1 day) than that of the pre-existing AChRs (t_1/2_ ≈ 5 days), even though these AChRs are intermingled in the same postsynaptic membrane. More importantly, it showed that the recycled receptors are continually inserted into the postsynaptic membrane and contribute to the postsynaptic receptor density. However, what remains unclear are (i) the proportion of the recycled receptor pool that derives from pre-existing or previously recycled AChRs, (ii) how many times receptors can recycle back to the membrane before being degraded, and (iii) why recycled AChRs are turned over more rapidly than pre-existing AChRs.

Because the extrasynaptic AChRs play an important role in maintaining synaptic AChR density [33,39], it was important to know about the metabolic stability of this receptor pool. Depending on the experimental design and the use of fluorescent or radioactive BTX ligand, it was reported that extrajunctional (nonsynaptic AChRs) turn over rapidly with a half-life in the membrane of approximately one day compared to junctional receptors that are much more stable (half-life that ranges between 9–14 days) [27,28,29,33,40,41,42]. It will be interesting to know about the mechanisms that underlie the rapid turnover rate of AChRs in the extrajunctional area of innervated and active muscle cells. 

The metabolic stability of aneural AChRs on developing cultured muscle cells was also extensively studied. Depending on cell type and experimental design, it was found that AChRs turned over rapidly at rates (half-life) ranging from 7 to 24 h. However, one must acknowledge that most of these studies have used either fluorescently tagged BTX or radio-labeled bungarotoxin, which it does not distinguish between diffuse and clustered receptors and did not take into account the pool of diffuse receptors that are able to laterally migrate into the receptor clusters over time, and that the estimate of receptor half-life is derived by pooling together all receptors from the entire population of cells [43,44,45,46]. However, when lateral migration of AChRs was taken into account, the half-life of AChRs at clusters was considerably low (t_1/2_ ≈ 4.5 h) [47]. In the cultured embryonic muscles [48,49] or organ cultures of muscles from newborn rats, it was also shown that AChRs turn over rapidly, similar to extrajunctional receptors in adult muscles [29,50]. However, in other experiments that used organ cultures from neonatal rat diaphragm muscles, it was demonstrated that junctional AChRs are degraded slowly, similar to the rates found in adult junctional receptors [50]. The discrepancy between the above results remains unclear.

At developing neuromuscular junction in living mice, when changes in fluorescently labeled AChRs at the same synapses were monitored over time, it was estimated that the half-life of AChRs is quite short during the first week after birth (*t*_1/2_ ≈ 26 h) and as synapses mature AChRs turn over more slowly [17]. Similarly, the fast turnover rate of junctional AChRs (t_1/2_ = 32 h) was also observed in chicken muscles one week after hatching, which considerably slowed (t_1/2_ ≥ 5 days) three weeks later [51]. 

## 3. The Effect of Synaptic Activity on the Metabolic Stability of AChR Pools

Previous studies have shown that muscle activity regulates the metabolic stability of AChRs in the postsynaptic membrane. In the absence of muscle activity following surgical denervation or pharmacological agents-induced paralysis, the turnover rate of AChRs was found to be considerably more rapid than the turnover rate in active muscles. For instance, at surgically denervated NMJs, the half-life of AChRs was significantly reduced from ∼ 9–14 days to 1–3 days [37,52,53,54]. A similar increase in the turnover of AChRs was observed in long-term inactivity of the innervated muscle caused by a tetrodotoxin cuff on the nerve [55]. In addition, at chronically blocked NMJs with either bungarotoxin or curare, the turnover rate of AChRs was also significantly increased with a half-life of hours [32]. Likewise, at neuromuscular diseases such as the autoimmune disease myasthenia gravis or muscles treated with IgG from myasthenia patients, the rate of AChRs degradation was significantly increased, leading to a decrease in AChR density and a reduction in synaptic folds [34,56,57,58,59]. A recent report also showed that in high-fat diet-induced obese male mice, the turnover rate of AChR significantly increased and the postsynaptic AChR density was considerably reduced, leading to a significant loss of postsynaptic receptor regions [60]. Of note, these obesity-related synaptic alterations were not seen in normal mice [60].

**Figure 1:**
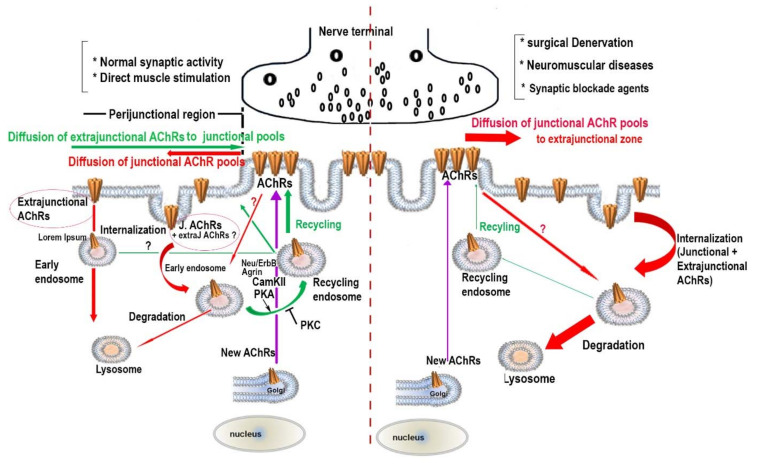
The metabolic stability of nAChRs at the functional and impaired peripheral cholinergic neuromuscular junction. A schematic diagram shows possible ways by which AChR pools (junctional and extrajunctional) are removed from and inserted into the neuromuscular junction under normal and pathological situations. Possible signaling molecules that mediate the recycling of AChRs are also represented.

As described above, the maintenance of a high density of AChRs in the postsynaptic membrane involves at least two separate pathways: a receptor-recycling pathway and a new synthesized receptor pathway [37]. Because recycled and pre-existing AChR pools at the same synapse can be separately labeled with a distinct fluorophore, it was possible to examine the effect of muscle activity on the turnover rate of these receptor pools. In the absence of muscle activity following denervation, it was found that the half-life of the pre-existing receptors was ∼ 1.9 days (nearly two times faster than the half-life at innervated synapses) and the half-life of the recycled AChRs was ∼ 15 h (nearly twice as fast as the half-life of recycled AChRs at innervated synapses). Muscle denervation not only accelerated the turnover rate of receptor pools at the NMJ but it also depressed the recycling of AChRs to the synaptic membrane by promoting the targeting of internalized AChRs to degradation [38]. It also should be noted that in the absence of synaptic activity, the delivery of new receptors to NMJs continues but at a reduced rate [37]. However, it remains unclear why recycled AChRs are turned over more rapidly than pre-existing AChRs. It appears that motor innervation plays a crucial role not only in the tethering and stabilization of AChRs in the postsynaptic membrane but also in promoting the recycling of internalized AChRs into the synaptic membrane (Figure 1).

Several studies have shown that direct muscle stimulation alone was able to reversibly increase the metabolic stability of receptors at surgically denervated synapses during both early development and adulthood, and at blocked endplates with pharmacological agents [32,61,62,63,64,65]. For instance, in denervated active/stimulated muscles, the half-life of junctional AChRs was similar to innervated muscles (*t*_1__/2_ ≈ 13 days) when compared to the turnover rates of AChRs in inactive muscles (*t*_1/2_ ≈ 1–5 days). These data indicate that muscle activity *prevents* the denervation-induced decline of metabolic AChR stability. Interestingly, in denervated-stimulated muscles, the loss of end-plate membrane structure caused by denervation is also largely prevented [61]. Further studies have reported that direct muscle stimulation of innervated and denervated muscles can promote the translocation of internalized receptors from the internal pool into the postsynaptic membrane [66]. This process appears to be associated with an increase in intracellular calcium concentration through either ligand-gated, L-type channels, or directly from intracellular stores. Interestingly, when L-type channels are blocked or intracellular calcium is clamped with BAPTA-AM significantly, the stability of AChR was significantly decreased [64] and the recycling of internalized receptors was attenuated [66]. Based on these observations, it would be reasonable to suggest continuous muscle stimulation-induced contractions can be used as an effective rehabilitative approach for patients suffering with permanent long-term denervation and for patients who develop paralytic syndrome when they are treated with reversible neuromuscular blocking agents for long periods of time.

## 4. The Regulatory Signaling Molecules Involved in the Metabolic Stability of AChRs at the NMJ

Attempts to understand the regulatory signaling pathways involved in the endocytosis, recycling, and new synthesis of AChRs have been investigated. During the last decade, a series of reports have provided a list of molecules that are involved in the endocytosis of AChRs. These include the autophagic regulator protein SH3 GLB1, the atrophy-promoting E3 ubiquitin ligase (MuRF1), the NRG/ErbB signaling pathway, the activation of Rac1, and the phosphorylation of Src [67,68,69,70,71,72]. Similar to processes of AMPA receptor internalization and recycling in the CNS, endocytic vesicles containing AChRs and exocytic vesicles containing recycled AChRs are found to be separately located at different sites within the NMJ. For instance, it was found that vesicles containing endocytic Rab5 and AChRs inside muscles are located in distant areas from the NMJ, while vesicles containing AChRs and recycling markers Rab4 and Rab11 are located mostly in close vicinity to the NMJ [73]. These observations were consistent with earlier data showing that the peri-junctional region (several micrometers from the junction folds) is the site of receptor internalization [32]. 

The mechanisms involved in the recycling of AChRs into the postsynaptic membrane remain largely unknown. A few studies have shown that activities of PKC and PKA (two serine/threonine kinases), which are located in and around the neuromuscular junction, were found to be involved in the recycling of AChRs. Inhibition of PKC or stimulation of PKA promotes the recycling of internalized AChRs into synaptic sites, while stimulation of PKC or inhibition of PKA depresses the recycling of AChRs and accelerates the removal rate of receptors from the postsynaptic membrane [74]. It was suggested that PKA controls the recycling of AChRs through its interaction with myosin Va, a protein that plays an important role in the PKA positioning and tethering to the actin cytoskeleton in a postsynaptic microdomain [75]. In other studies, rapsyn was also described as an anchoring protein (AKAP) that links PKA to AChR-recycling vesicles [76]. Further studies have shown that the release of α-calcitonin gene-related peptide (CGRP) from motor neurons and sympathetic innervation of NMJs are essential for stimulating the production of cAMP and activation of PKA [77,78]. Interestingly, in denervated muscles pharmacological elevation of cytoplasmic cAMP and activation of PKA slow the turnover of nAChRs [79]. It appears that a fine balance between activation of PKC and inhibition of PKA is critical for the recycling, and insertion of new receptor clusters and the disassembly of pre-existing ones [74,80]. Interestingly, work by Bruneau et al. [38] showed that inhibition of tyrosine phosphatase activity caused the mistargeting of recycled AChRs specifically to extrasynaptic regions (outside of the usually sharp NMJ boundary) while pre-existing receptors remain intact within the NMJ [38]. The same observation has been made in the synapses of frog nerve/muscle co-cultures treated with tyrosine phosphatase inhibitors [81]. Of note, the phosphorylation of ADF/cofilin-mediated actin was also shown to be involved in the trafficking and targeting of AChRs to the cell surface, particularly to nascent postsynaptic sites [82].

The synaptic organizer agrin also has been shown to play an essential role in the recycling of nAChRs. Experiments on nerve-free AChR clusters induced by agrin in the extrasynaptic membrane in living rats showed that internalized AChRs were able to recycle back into the ectopic synaptic clusters where they intermingle with pre-existing and new receptors and that the extent of AChR recycling depended on the strength of the agrin stimulus [83]. 

The role of Ca^2+^/calmodulin-dependent kinase II in the control of the recycling of the nAChR has been investigated. In muscle cells, several CaMKII isoforms (CamkII βM, γ, δ, but not α isoform) are expressed and when intracellular calcium is chelated or CaMKII activity is inhibited, receptor recycling is depressed, altering the trafficking of receptors and the steady-state of the postsynaptic receptor density [17]. By increasing or decreasing intracellular calcium concentration, the muscle cell can up- or downregulate the cycling of AChR into the postsynaptic membrane. This process is mediated by CaMKII activity as blockade of the enzyme dramatically inhibits receptor recycling. These results establish a role for calcium and calcium-activated kinase, CaMKII in the recycling of receptors at the NMJ in vivo. Recent work has also shown that a muscle-specific nonkinase anchoring protein (αkap) encoded within the Camk2a gene plays a critical role in promoting the stability of AChR by a ubiquitin-dependent mechanism. The Knockdown of αkap with shRNA in cultured muscle cells significantly enhanced the degradation of AChR, leading to fewer and smaller AChR clusters on the surface of differentiated C2C12 myotubes [84], and in vivo significantly enhancing the turnover rate of AChR, and altering the structural integrity of the NMJ [85]. 

Previous studies have shown that neuregulins signal through the ErbB family of tyrosine kinase receptors is not required for synapse-specific expression of genes by subsynaptic nuclei of the mouse NMJ [86]. However, the analysis of the turnover rate of AChRs in mice deficient in *erbb2* and *erbb4* selectively in muscles (*erbb2/4^−/−^*) showed that the half-life of AChRs was significantly decreased, specifically, the recycled receptor pool. In addition, as the contribution of recycled and new receptors to the receptor pools at synaptic sites is roughly equal, it appears that the destabilization of the recycled receptor pool was instrumental in the lowering of the density of AChRs locally and thus the disassembly of the postsynaptic apparatus observed at the NMJ of these mice [69]. Further experiments showed that the destabilization of AChRs is mediated by α-dystrobrevin [a component of the dystrophin glycoprotein complex (DGC) that is a substrate for ErbB receptor tyrosine kinases] as evidenced by its dephosphorylated state in the absence of NRG/ErbB signaling. The role of isoforms of tyrosine phosphorylated α-dystrobrevin in the stability of AChRs and the maintenance of the overall structural integrity of the synapse has been previously studied. In the absence of phosphorylated α-dystrobrevin, the density as well as the turnover rates were significantly altered. Likewise, in mice deficient in α-syntrophin (another component of the DGC), the turnover rate of AChR is significantly increased (3–4 days compared to 9–14 days in wildtype). Importantly, the number of recycled AChR is also reduced in mice deficient in α-syntrophin and α-dystrobrevin, which may account for the reduced AChR densities and numbers in these mutant mice [17]. While there is no direct link between α-syntrophin or dystrobrevin and AChRs, it is clear that the loss of either α-syntrophin or α-dystrobrevin drastically impairs the postsynaptic receptor density [87]. However, not all components of DGC are involved in the regulation of the metabolic stability of AChRs. For instance, in dystrophin-deficient NMJs, the turnover rate is similar to wild type [33]. 

## 5. Concluding Remarks

The picture emerging from recent work is that nAChRs in the postsynaptic apparatus are highly dynamic despite the overall structural stability of the neuromuscular junction e (Figure 1). Identifying and understanding signals triggered by muscle action potentials and nerves that are involved in the regulation of AChR dynamics (removal, insertion of new synthesized, and recycling) should provide important insights not only into mechanisms that control the structural integrity of the NMJ but also for neuromuscular diseases in which the density/number and turnover rates of AChRs are compromised. It is also equally important to understand the regulatory mechanism involved in the trafficking of AChRs from their assembly in the endoplasmic reticulum to their insertion into the cell membrane, and how reducing the endocytosis or enhancing the recycling of AChRs could be beneficial for many neuromuscular diseases where the density/number of synaptic AChRs is compromised. 

## Data Availability

Not applicable.
